# aMMP‐8 self‐testing and self‐administered questionnaires for periodontitis screening: A diagnostic trial in a Chinese population

**DOI:** 10.1002/jper.70032

**Published:** 2025-12-10

**Authors:** Yu Xie, Xiaoyu Yu, Mengning Bi, Hairui Li, Yuan Li, Maurizio S. Tonetti

**Affiliations:** ^1^ Shanghai PerioImplant Innovation Center Institute for Oral Craniofacial and Sensory Research Ninth People's Hospital Shanghai Jiao Tong University School of Medicine Shanghai China; ^2^ National Clinical Research Center of Oral Diseases and National Center of Stomatology College of Stomatology Shanghai Jiao Tong University School of Medicine Shanghai China; ^3^ Department of Oral Implantology Ninth People's Hospital Shanghai China; ^4^ European Research Group on Periodontology (ERGOPerio) Genova Italy

**Keywords:** diagnosis, matrix metalloproteinase‐8, periodontitis, self‐report

## Abstract

**Background:**

Limited awareness, clinical barriers, and persistent high prevalence make large‐scale detection of periodontitis challenging, creating demand for simple, affordable, and population‐level screening strategies outside traditional dental settings. This study evaluates the diagnostic performance of self‐test activated matrix metalloproteinase‐8 (aMMP‐8) for identifying periodontitis and assesses the feasibility of a fully self‐reported screening model compared with models based on quantitative aMMP‐8 point‐of‐care test (POCT) results.

**Methods:**

In this cross‐sectional diagnostic study, we evaluated the diagnostic performance of three index tests: self‐performed or POCT aMMP‐8 alone on an oral rinse sample, or in combination with a questionnaire. Periodontitis status was determined using radiographic bone loss according to the 2017 classification, as stages III–IV or stages II–IV. Binary logistic regression models were developed using least absolute shrinkage and selection operator regularization and 5‐fold cross‐validation. Model performance was assessed by area under the receiver operating characteristic curve (AUROC), sensitivity, specificity, and accuracy.

**Results:**

A total of 566 participants were included, and 41.7% were diagnosed with stages II–IV periodontitis. Both self‐test and quantitative aMMP‐8 results were significantly associated with periodontitis. In univariable models, self‐test aMMP‐8 showed lower AUROC than quantitative results. However, adding questionnaires and demographic factors in multivariable models improved the diagnostic performance of self‐test and minimized the gap between the two. The final multivariable models showed comparable performance for self‐test aMMP‐8‐based (AUROC = 0.955) and quantitative aMMP‐8‐based (AUROC = 0.957) models in stages III–IV periodontitis prediction, with similar results for stages II–IV periodontitis.

**Conclusion:**

This study showed that a self‐test aMMP‐8‐based multivariable diagnostic model can perform similarly to a quantitative aMMP‐8 POCT‐based one. Once further developed and validated, a fully self‐reported diagnostic model can provide a potential tool for periodontitis screening.

**Plain language summary:**

This study examined whether a simple, self‐administered mouth rinse test could aid in identifying individuals with advanced gum disease. Over 550 adults participated, and approximately 42% were found to have severe gum disease. We discovered that the self‐test alone was less accurate than a more detailed machine‐based quantitative reading. However, when combined with a questionnaire about dental health and basic personal information, the accuracy of detection improved a lot. These combined methods performed well and were similar to using only the machine results. Our results suggest that a simple screening approach, which includes a self‐test, a questionnaire, and personal details, could be an inexpensive and easy‐to‐use tool for community health screenings to identify severe gum disease early. Further research is needed to test this method in various populations and confirm its effectiveness in real‐world settings for identifying gum disease and promoting early dental care.

## 1 INTRODUCTION

Periodontitis is a common chronic non‐communicable disease^1^ affecting oral health,^2^ general health comorbidities,^3^, ^4^ and quality of life.^5^, ^6^ Despite its harm and severity, public awareness remains limited.^7^, ^8^ Meanwhile, symptoms such as bleeding gums, swelling, or tooth mobility are often overlooked.^9^, ^10^ This neglect contributes to a high and persistent prevalence of the disease, placing additional strain on healthcare systems.^11^, ^12^ Therefore, there is an urgent need for accurate, effective, and affordable screening tools for periodontitis, especially for deployment in large‐scale public health and primary care environments.

Full‐mouth clinical examinations are the gold standard for diagnosing periodontitis,^13^ but their use is limited by time, costs, and the need for specialized training.^14^, ^15^ Simplified methods like those based on the Community Periodontal Index of Treatment Needs (CPITN) from the WHO also face issues that hinder their accuracy and adoption.^16^, ^17^ Radiographic tools are often used in practice, despite their low sensitivity for early detection^18^ and issues like image distortion.^15^ Orthopantomograms (OPGs) help assess marginal bone loss in stages II–IV of periodontitis,^19^ but the WHO advises reducing radiographic exams due to concerns over radiation exposure and health.^20^, ^21^

In addition, even in many high‐income countries, only a minority of the population regularly access dental services.^22^, ^23^ In this context, finding alternative approaches for periodontal screening is crucial. Nonclinical diagnostic models have emerged as promising tools for periodontitis screening, as they are noninvasive, cost‐effective, and rapid.^24^, ^25^, ^26^, ^27^, ^28^, ^29^, ^30^ These models typically rely on two key components: self‐reported questionnaires and biomarkers. The self‐reported questionnaire from the Centers for Disease Control and Prevention and the American Academy of Periodontology (CDC/AAP) has been widely evaluated^31^ and showed fair diagnostic performance in detecting severe periodontitis.^28^, ^32^ However, applications of validated versions in European and Chinese samples have shown lower accuracy,^25^, ^33^ perhaps due to cultural biases or population differences.

Among biomarkers, active matrix metalloproteinase‐8 (aMMP‐8) is a key enzyme in tissue degradation during periodontitis.^26^, ^27^, ^34^ With the advent of point‐of‐care testing (POCT), quantitative aMMP‐8 detection has become feasible in chairside settings, and our prior studies confirmed its moderate diagnostic ability for periodontitis.^34^, ^35^ Importantly, combining quantitative aMMP‐8 POCT with self‐reported questionnaires has shown superior diagnostic performance compared with either method alone.^24^ However, the accuracy of aMMP‐8 self‐testing, a simpler, more accessible alternative that a patient can perform at home, has not yet been explored.

The general hypothesis of this study is that self‐test aMMP‐8 results, either on their own or in combination with certain self‐reported nonclinical features assessed by questionnaire and related to periodontal health or disease, can function as a screening tool for detecting periodontitis among a Chinese population.

The specific objectives include: (1) Comparing the diagnostic accuracy of dichotomous aMMP‐8 self‐test results with quantitative assessments obtained using a POCT reader to identify individuals with periodontitis; (2) Developing and testing multivariable diagnostic models that incorporate either self‐test or quantitative aMMP‐8 readings, combined with the modified CDC/AAP questionnaire and demographic factors, to predict stages III–IV or II–IV of periodontitis; and (3) Comparing the diagnostic performance of self‐test models with those obtained through POCT to evaluate the feasibility of using a self‐test screening approach for periodontitis.

## 2 MATERIALS AND METHODS

### 2.1 Study design and population

This cross‐sectional diagnostic study recruited a sample of consecutive patients seeking dental care at the Ninth People's Hospital, Shanghai, from July 2023 to July 2024. The inclusion criteria were dentate adults (aged ≥18 years) who were willing to provide written informed consent. Exclusion criteria included pregnancy, antibiotic use within the past 3 months, or receipt of professional periodontal treatment within the previous 12 months. The study protocol was registered on ClinicalTrials.gov (NCT03928080). Ethical approval was obtained from the Research Ethics Committee of Shanghai Ninth People's Hospital (SH9H‐2021‐T408‐3). This study followed the Standards for Reporting Diagnostic Accuracy (STARD) guidelines.^36^

### 2.2 Sample size estimation

The sample size was estimated using a one‐sample proportion test based on models for periodontitis screening. Reference sensitivity and specificity values were drawn from a systematic review of quantitative aMMP‐8 test performance (60% sensitivity and 82% specificity).^37^ In the absence of previous work on multimodal approaches, for this study the target performance was set at 70% sensitivity and 85% specificity, considering both the reported performance of aMMP‐8 and the previously reported performance of the CDC/AAP questionnaire under the 2017 classification.^33^ Assuming a significance level of 5%, statistical power of 85%, a 10% rate of missing data, and an estimated 50% prevalence of periodontitis in mainland China,^1^ the required sample size was calculated as 565 participants. All sample size estimations were conducted using standard statistical software*.

### 2.3 Index tests of predictors

The study included three index tests: the aMMP‐8 dichotomous self‐test, its point‐of‐care quantitative reading, and an expanded CDC/AAP questionnaire. Demographics, lifestyle factors, and health conditions, including smoking, alcohol consumption, and systemic diseases, were all self‐reported on a tablet computer. A trained nurse measured participants' height and weight to calculate body mass index (BMI).

For the aMMP‐8 test, oral rinse samples were used. Participants were required to avoid eating, drinking, brushing, or using mouthwash for at least 1 h before testing. The test was performed according to the manufacturer's instructions, and results were assessed both by self‐reporting and with a digital device.^24^ For the self‐test, participants received instructions by a trained nurse. Each participant conducted a 30‐second rinse with a commercial test kit†. The rinse was then filtered, and three to four drops were applied to the test strip. Following a 5‐minute incubation, participants visually assessed the paper strip, following specific instructions for detecting one or two blue bands on the strip, and reported the results as positive (two bands) or negative (one band). The same strip was then immediately read using a digital immunoassay test system‡, operated by the trained nurse, which provided quantitative aMMP‐8 concentrations. The obtained values were normalized by dividing by the number of teeth present (aMMP‐8/NTP), as previous research indicated higher accuracy measures.^35^

Participants were asked to fill out the expanded CDC/AAP questionnaire by themselves before the radiographic assessment without any help from others. The CDC/AAP questionnaire has been widely used worldwide,^31^ and the Chinese version of the original CDC/AAP questionnaire has been previously developed and validated.^24^, ^33^ This study included five additional questions exploring Q9‐“bleeding gums,” Q10‐“frequency of gum bleeding,” Q11‐“difficulty in chewing,” Q12‐“decrease in food intake,” and Q13‐“change in the type of food” based on previous studies of gingival bleeding and masticatory dysfunction.^30^, ^38^, ^39^ The original and Chinese translations of the questions are shown in Table S1 in the online *Journal of Periodontology*.

### 2.4 Radiographic assessment and case definition

**FIGURE 1:**
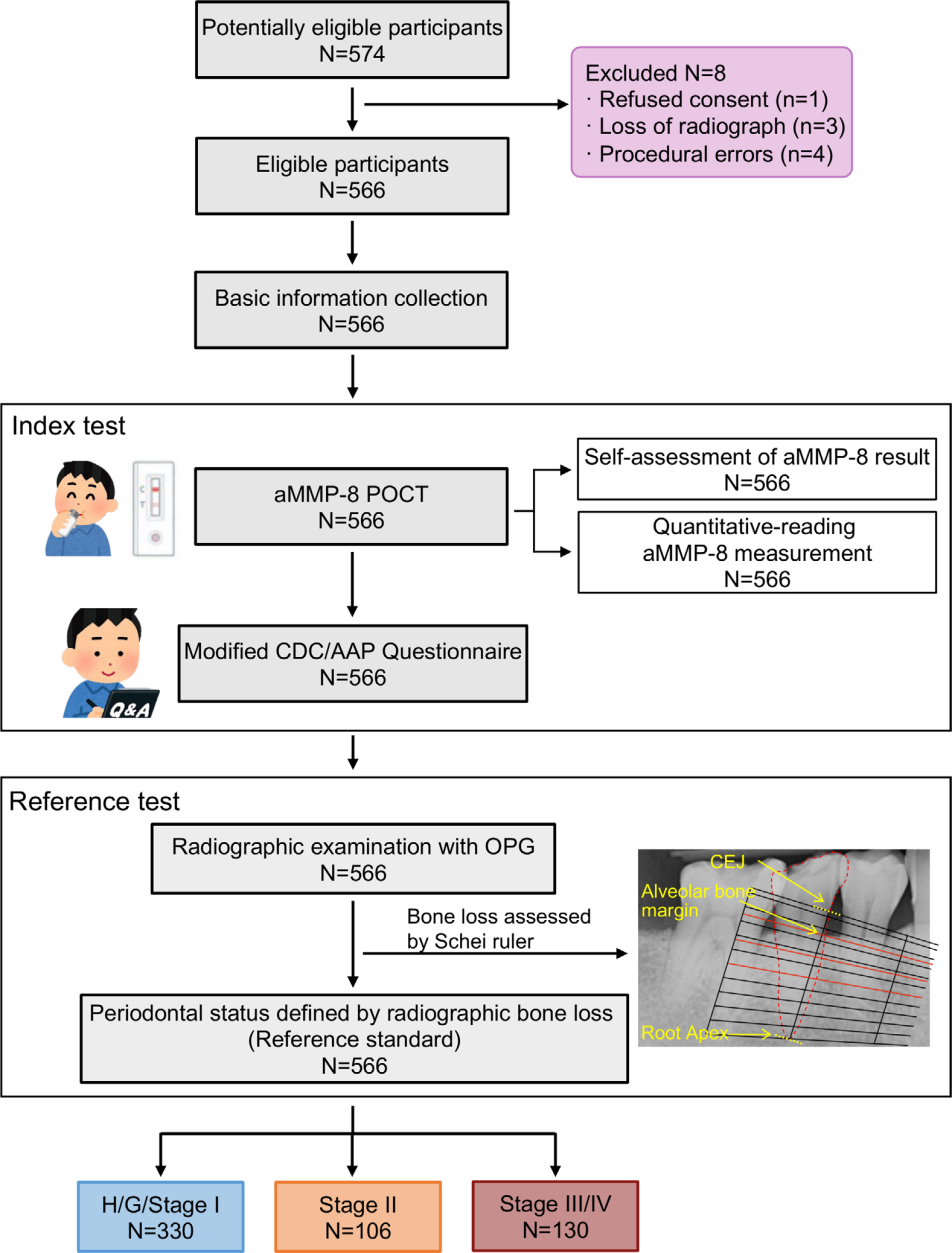
Study procedure flowchart. The figure highlights study procedures, illustrating participant inclusion, index tests and reference test. aMMP‐8, activated matrix metalloproteinase‐8; CDC/AAP, Centers for Disease Control and Prevention‐American Academy of Periodontology; CEJ, cemento‐enamel junction; OPG, orthopantomogram; POCT, point‐of‐care test.

All participants underwent an OPG screening following hospital policy. The periodontal case definition was established based on the radiographic assessment of marginal alveolar bone loss using a modified Schei ruler,^40^ which has reference marks at 15%, 33%, and 50% of root length to assist in visually evaluating bone loss according to the accepted severity staging criteria^41^, ^42^ (Figure 1).

Due to the inherent limitations of radiographic diagnosis, participants were classified into three categories based on bone loss patterns according to the 2017 Classification^13^: (i) No detectable bone loss or bone loss < 15% of root length on all teeth were defined as Health/Gingivitis/Stage I Periodontitis (H/G/Stage I); (ii) Bone loss of 15%–33% in at least two nonadjacent interproximal sites were defined as Stage II Periodontitis (Stage II); (iii) Bone loss > 33% in at least two nonadjacent interproximal sites were defined as Stages III–IV Periodontitis (Stages III–IV). Based on the radiographic information, it was not possible to discriminate between stage III and stage IV periodontitis.

Two trained and calibrated examiners (YX, XYY), blinded to the index test results, independently assessed radiographic bone loss and assigned periodontal status to all participants. The reproducibility for case definitions was 0.92 (YX) and 0.91 (XYY). Disagreements were resolved through discussion with the reference examiner (MST).

### 2.5 Data analysis

Statistical analyses were conducted using standard statistical software§. Descriptive statistics were used to summarize demographic characteristics and index test results across different groups based on periodontal status. The normality of continuous variables was assessed; non‐normally distributed data were reported as median (interquartile range, IQR) and compared using the Kruskal–Wallis test. When significant differences were identified among groups, pairwise post hoc comparisons were conducted using Dunn test with Bonferroni correction. Categorical variables were presented as counts (frequencies) and analyzed using the Chi‐square test.

Binary logistic regression was employed to evaluate associations between index test results and periodontal status. Two outcome classifications were analyzed: (a) “stages III–IV periodontitis” compared with the combined group of “health/gingivitis/stage I periodontitis” and “stage II periodontitis”; and (b) the combined group of “stage II periodontitis” and “stages III–IV periodontitis” compared with “health/gingivitis/stage I periodontitis.” After merging infrequent response categories from the self‐test questionnaire,^25^ univariable logistic regression was used to estimate odds ratios (ORs) and 95% confidence intervals (CIs) for individual predictors.

Diagnostic models were developed based on either self‐test aMMP‐8 results or quantitative aMMP‐8 readings, along with their combinations with the modified CDC/AAP questionnaire and demographic/risk factors. Multivariable models were constructed using the least absolute shrinkage and selection operator (LASSO)‐regularized logistic regression to select relevant predictors while reducing overfitting and multicollinearity. Internal validation was performed using stratified 5‐fold cross‐validation. The optimal cut‐off point for each model was determined based on the maximum sum of sensitivity and specificity.^25^

Model performance was evaluated using aggregated test data from cross‐validation, looking at sensitivity, specificity, accuracy, and the area under the receiver operating characteristic curve (AUROC). Sensitivity and specificity were categorized as low (< 60%), moderate (60%–79%), or high (≥80%).^43^ AUROC values were interpreted as low (0.50–0.70), moderate (0.71–0.90), or high (> 0.90).^44^ A *p*‐value of less than 0.05 was considered statistically significant.

## 3 RESULTS

Of 574 potentially eligible individuals, 566 participants were included in this study (refer to Figures 1 and S1 [in the online *Journal of Periodontology*] for the STARD diagram). According to the 2017 classification system, 58.3% of participants had periodontal health, gingivitis, or stage I periodontitis; 18.7% were stage II periodontitis; and 23.0% were stages III–IV periodontitis. The demographic and medical characteristics of the study population are summarized in Table 1. The median age of the participants was 30 years (IQR 22–47). Additionally, 7.4% of participants reported being current smokers and 1.2% had diabetes. Most participants (88.1%) had a moderate‐to‐high level of education, defined as having completed junior college or above.

**TABLE 1:** Demographic and medical characteristics of the study population in whole population (*n* = 566) and in different periodontal status.

	All (*n* = 566)	Health/Gingivitis/ Stage I periodontitis [radiographic bone loss < 15%] (*n* = 330)	Stage II periodontitis [radiographic bone loss between 15% and 33%] (*n* = 106)	Stages III–IV Periodontitis [radiographic bone loss > 33%] (*n* = 130)	*p*‐value
**Age**	30 (22–47)	23 (21–28)	41 (32–52)	55 (47–61)	<0.001
**Sex**					<0.001
Male	217 (38.3%)	98 (29.7%)	42 (39.6%)	77 (59.2%)	
Female	349 (61.7%)	232 (70.3%)	64 (60.4%)	53 (40.8%)	
**Education level**					<0.001
No education/Elementary school/Junior high school	22 (3.9%)	3 (0.9%)	8 (7.5%)	11 (8.5%)	
Senior high school /Technical secondary school	45 (8%)	15 (4.5%)	10 (9.4%)	20 (15.4%)	
Junior college/Bachelor	342 (60.4%)	196 (59.4%)	65 (61.3%)	81 (62.3%)	
Master/Doctor	157 (27.7%)	116 (35.2%)	23 (21.7%)	18 (13.8%)	
**Family income** ^a^					0.020
Low income	112 (19.8%)	68 (20.6%)	17 (16%)	27 (20.8%)	
Medium income	289 (51.1%)	175 (53%)	45 (42.5%)	69 (53.1%)	
High income	124 (21.9%)	59 (17.9%)	36 (34%)	29 (22.3%)	
Don't know	41 (7.2%)	28 (8.5%)	8 (7.5%)	5 (3.8%)	
**Number of teeth present (NTP)**	27 (25–28)	28 (27–28)	27 (25–28)	25 (21–27)	<0.001
**BMI (kg/m^2^)**	22.5 (20.4–24.8)	22.0 (19.9–24.4)	23.0 (20.6–25)	23.7 (21.9–25.9)	<0.001
**Diabetes status** ^b^					0.003
No	559 (98.8%)	330 (100.0%)	104 (98.1%)	125 (96.2%)	
Yes	7 (1.2%)	0 (0.0%)	2 (1.9%)	5 (3.8%)	
**Smoking status**					<0.001
Never	493 (87.1%)	312 (94.5%)	89 (84%)	92 (70.8%)	
Former smoker	31 (5.5%)	6 (1.8%)	6 (5.7%)	19 (14.6%)	
Current smoker	42 (7.4%)	12 (3.6%)	11 (10.4%)	19 (14.6%)	
Heavy smoker^c^	18 (3.2%)	2 (0.6%)	6 (5.7%)	10 (7.7%)	
**Alcohol consumption** ^d^					0.081
No	229 (40.5%)	140 (42.4%)	42 (39.6%)	47 (36.2%)	
Yes	333 (58.8%)	190 (57.6%)	63 (59.4%)	80 (61.5%)	
Former consumption	4 (0.7%)	0 (0.0%)	1 (0.9%)	3 (2.3%)	

Abbreviation: BMI, body mass index.

^a^ For family income, low income means 0–5000 Renminbi (RMB, Chinese Yuan) per person per month, medium income means 5000–15000 RMB, and high income means > 15000 RMB. Conversion rate: 1 RMB ≈ 0.14 USD (September 2025).

^b^ Diabetes status was recorded based on participants’ self‐reporting.

^c^ Heavy smoker means self‐reported smoking more than 10 cigarettes per day.

^d^ For alcohol consumption, “No” refers to individuals who self‐reported to have never consumed alcoholic beverages, “Yes” refers to those who currently consume alcoholic beverages (regardless of frequency or quantity), and “Former consumption” refers to individuals who previously consumed alcohol but no longer do so.

### 3.1 Univariable associations of aMMP‐8 results and modified CDC/AAP questionnaire responses with different periodontal status

The results of the self‐test and quantitative aMMP‐8 levels are presented in Table 2. The thresholds of quantitative aMMP‐8 levels were established by maximizing the combined sensitivity and specificity, known as the Youden Index. Both aMMP‐8 test results displayed an increasing trend correlated with the severity of periodontitis. The proportion of participants with a positive aMMP‐8 self‐report showed an increasing trend consistent with greater periodontitis severity. Specifically, the self‐reported positive rate was 58.5% among individuals with health, gingivitis, or stage I periodontitis, increased to 67.0% in those with stage II periodontitis, and further rose to 79.2% in participants with stages III–IV periodontitis.

**TABLE 2:** Self‐report questionnaire responses and aMMP‐8 POCT test results across different periodontal status, and the diagnostic ability for different periodontal status evaluated by univariable logistic regression.

		According to periodontal status defined by radiographic bone loss					
Predictors	All (*n*=566)	Health/Gingivitis/Stage I Periodontitis (*n*=330)	Stage II Periodontitis (*n*=106)	Stages III‐IV Periodontitis (*n*=130)	*p*‐value	Odds ratio (stages III‐IV versus H/G/stages I‐II)	Odds ratio (stages II‐IV versus H/G/stage I)
**aMMP‐8 self‐report**					**<0.001**	2.49 (1.56–3.96)^a^ ***p* < 0.001**	1.99 (1.39–2.86)^a^ ***p* < 0.001**
Negative	199 (35.2%)	137 (41.5%)	35 (33.0%)	27 (20.8%			
Positive	367 (64.8%)	193 (58.5%)	71 (67.0%)	103 (79.2%)			
**aMMP‐8 value (ng/mL)**	19 (10–44)	11 (10–26)	30 (10–51)	50 (22–86)	**<0.001**	1.03 (1.02–1.03) ***p* < 0.001**	1.03 (1.03–1.04) ***p* < 0.001**
**aMMP‐8/NTP**	0.7 (0.4–1.7)	0.4 (0.4–1.0)	1.2 (0.4–1.8)	2.1 (1.0–3.5)	**<0.001**	2.19 (1.85–2.59) ***p* < 0.001**	2.63 (2.13–3.25) ***p* < 0.001**
**Q1. Do you think you might have gum disease? (n/%)**					**<0.001**	6.87 (4.41–10.69)^b^ ***p* < 0.001**	4.97 (3.46–7.16)^b^ ***p* < 0.001**
Yes	223 (39.4%)	79 (23.9%)	48 (45.3%)	96 (73.8%)			
No	173 (30.6%)	131 (39.7%)	31 (29.2%)	11 (8.5%)			
Don't know	170 (30.0%)	120 (36.4%)	27 (25.5%)	23 (17.7%)			
**Q2. Overall, how would you rate the health of your teeth and gum? (n/%)**					**<0.001**	4.74 (3.05–7.37)^c^ ***p* < 0.001** 11.63 (5.01–27.00)^d^ ***p* < 0.001**	5.73 (3.62–9.08)^c^ ***p* < 0.001** 7.73 (4.70–12.73)^d^ ***p* < 0.001**
Poor	116 (20.5%)	30 (9.1%)	30 (28.3%)	56 (43.1%)			
Fair	273 (48.2%)	152 (46.1%)	58 (54.7%)	63 (48.5%)			
Good	120 (21.2%)	102 (30.9%)	15 (14.2%)	3 (2.3%)			
Very good	34 (6.0%)	31 (9.4%)	0 (0.0%)	3 (2.3%)			
Excellent	9 (1.6%)	9 (2.7%)	0 (0.0%)	0 (0.0%)			
Don't know	14 (2.5%)	6 (1.8%)	3 (2.8%)	5 (3.8%)			
**Q3a. Have you ever had treatment for gum disease, such as supragingival scaling? (n/%)**					**<0.001**	2.44 (1.5–3.95)^b^ ***p* < 0.001**	2.39 (1.63–3.50)^b^ ***p* < 0.001**
Yes	387 (68.4%)	201 (60.9%)	80 (75.5%)	106 (81.5%)			
No	175 (30.9%)	126 (38.2%)	26 (24.5%)	23 (17.7%)			
Don't know	4 (0.7%)	3 (0.9%)	0 (0.0%)	1 (0.8%)			
**Q3b. Have you ever had treatment for gum disease, such as subgingival scaling and root planing, sometimes called “deep cleaning”? (n/%)**							
Yes	120 (21.2%)	38 (11.5%)	23 (21.7%)	59 (45.4%)	**<0.001**	5.11 (3.29–7.92)^b^ ***p* < 0.001**	4.09 (2.66–6.30)^b^ ***p* < 0.001**
No	408 (72.1%)	268 (81.2%)	77 (72.6%)	63 (48.5%)			
Don't know	38 (6.7%)	24 (7.3%)	6 (5.7%)	8 (6.2%)			
**Q4. Have you ever had any teeth become loose on their own without an injury? (n/%)**					**<0.001**	14.38 (9.05–22.83)^b^ ***p* < 0.001**	6.82 (4.47–10.42)^b^ ***p* < 0.001**
Yes	149 (26.3%)	38 (11.5%)	21 (19.8%)	90 (69.2%)			
No	351 (62.0%)	244 (73.9%)	74 (69.8%)	33 (25.4%)			
Don't know	66 (11.7%)	48 (14.5%)	11 (10.4%)	7 (5.4%)			
**Q5. Have you ever been told by a dental professional that you lost bone around your teeth? (n/%)**					**<0.001**	8.42 (5.40–13.15)^b^ ***p* < 0.001**	6.39 (4.08–9.99)^b^ ***p* < 0.001**
Yes	128 (22.6%)	32 (9.7%)	24 (22.6%)	72 (55.4%)			
No	356 (62.9%)	252 (76.4%)	65 (61.3%)	39 (30.0%)			
Don't know	82 (14.5%)	46 (13.9%)	17 (16%)	19 (14.6%)			
**Q6. During the past 3 months, have you noticed a tooth that doesn't look right? (n/%)**					**<0.001**	3.01 (2.02–4.51)^b^ ***p* < 0.001**	2.30 (1.62–3.26)^b^ ***p* < 0.001**
Yes	203 (35.9%)	92 (27.9%)	38 (35.8%)	73 (56.2%)			
No	325 (57.4%)	212 (64.2%)	62 (58.5%)	51 (39.2%)			
Don't know	38 (6.7%)	26 (7.9%)	6 (5.7%)	6 (4.6%)			
**Q7. Aside from brushing your teeth with a toothbrush, in the last 7 days, how many times did you use dental floss or any other device to clean between your teeth? (n/%)**							
No	228 (41.9%)	144 (44.3%)	34 (34.0%)	50 (42.0%)	0.241	0.87 (0.58–1.28)^b^ *p* = 0.472	1.10 (0.79–1.54)^b^ *p* = 0.578
1‐7 times/week	259 (47.6%)	153 (47.1%)	53 (53.0%)	53 (44.5%)			
≥8 times/week	57 (10.5%)	28 (8.6%)	13 (13.0%)	16 (13.4%)			
Don't know	22 (3.9%)	5 (1.5%)	6 (5.7%)	11 (8.5%)			
**Q8. Aside from brushing your teeth with a toothbrush, in the last 7 days, how many times did you use mouthwash or other dental rinse product that you use to treat dental disease or dental problems? (n/%)**							
No	433 (79.3%)	260 (80.2%)	74 (74.0%)	99 (81.1%)	0.663	0.83 (0.50–1.37)^b^ *p* = 0.461	1.09 (0.72–1.65)^b^ *p* = 0.688
1‐7 times/week	101 (18.5%)	58 (17.9%)	23 (23.0%)	20 (16.4%)			
≥8 times/week	12 (2.2%)	6 (1.9%)	3 (3.0%)	3 (2.5%)			
Don't know	20 (3.5%)	6 (1.8%)	6 (5.7%)	8 (6.2%)			
**Q9. Have you ever had bleeding gums when brushing your teeth? (n/%)**					0.807	1.40 (0.80–2.43)^b^ *p* = 0.235	1.22 (0.78–1.91)^b^ *p* = 0.385
Yes	468 (82.7%)	269 (81.5%)	87 (82.1%)	112 (86.2%)			
No	84 (14.8%)	53 (16.1%)	16 (15.1%)	15 (11.5%)			
Don't know	14 (2.5%)	8 (2.4%)	3 (2.8%)	3 (2.3%)			
**Q10. How often did you have bleeding gums when brushing your teeth? (n/%)**					**0.048**	3.53 (1.38–9.03)^e^ ***p* = 0.008** 1.35 (0.91–2.01)^f^ *p* = 0.132	2.50 (1.34–4.68)^e^ ***p* = 0.004** 1.65 (1.18–2.31)^f^ ***p* = 0.004**
Never	59 (10.4%)	45 (13.6%)	9 (8.5%)	5 (3.8%)			
Seldom	222 (39.2%)	136 (41.2%)	34 (32.1%)	52 (40.0%)			
Occasionally	202 (35.7%)	107 (32.4%)	44 (41.5%)	51 (39.2%)			
Fairly often	57 (10.1%)	28 (8.5%)	14 (13.2%)	15 (11.5%)			
Very often	26 (4.6%)	14 (4.2%)	5 (4.7%)	7 (5.4%)			
**Q11. Do you experience difficulty chewing? (n/%)**					**<0.001**	9.52 (5.84–15.52)^b^ ***p* < 0.001**	6.59 (3.91–11.10)^b^ ***p* < 0.001**
Yes	94 (16.6%)	21 (6.4%)	14 (13.2%)	59 (45.4%)			
No	472 (83.4%)	309 (93.6%)	92 (86.8%)	71 (54.6%)			
Don't know	0 (0.0%)	0 (0.0%)	0 (0.0%)	0 (0.0%)			
**Q12. Has the amount of food you usually eat decreased in the last year because of chewing problems? (n/%)**					**<0.001**	7.02 (3.59–13.72)^b^ ***p* < 0.001**	4.22 (2.07–8.61)^b^ ***p* < 0.001**
Yes	41 (7.2%)	11 (3.3%)	4 (3.8%)	26 (20.0%)			
No	525 (92.8%)	319 (96.7%)	102 (96.2%)	104 (80.0%)			
Don't know	0 (0.0%)	0 (0.0%)	0 (0.0%)	0 (0.0%)			
**Q13. Did you need to change the type of food you eat because of chewing problems? (n/%)**					**<0.001**	8.69 (5.36–14.08)^b^ ***p* < 0.001**	5.49 (3.33–9.05)^b^ ***p* < 0.001**
Yes	95 (16.8%)	24 (7.3%)	13 (12.3%)	58 (44.6%)			
No	471 (83.2%)	306 (92.7%)	93 (87.7%)	72 (55.4%)			
Don't know	0 (0.0%)	0 (0.0%)	0 (0.0%)	0 (0.0%)			

*Note*: *p* < 0.05 were labeled as bold. The questionnaire is derived from the CDC/AAP questionnaire; odds ratio were present as odds ratio (95% confidence interval).

Abbreviations: aMMP‐8: activated matrix metalloproteinase‐8; POCT: point‐of‐care test; NTP: number of teeth present; OR: odds ratio.

^a^ Reference is the “Negative” self‐reports.

^b^ Reference is the combination of “No” + “Don't know” answers.

^c^ Reference is the combination of answers “Excellent” + “Very good” + “Good” + “Fair” + “Don't know”.

^d^ Reference is the combination of answers “Excellent” + “Very good” + “Good”.

^e^ Reference is the combination of answers “Very often” + “Fairly often” + “Occasionally” + “Seldom”.

^f^ Reference is the combination of answers “Very often” + “Fairly often” + “Occasionally”.

Similarly, both the overall aMMP‐8 values and the aMMP‐8 values normalized by the number of teeth (aMMP‐8/NTP) showed significant increases in participants with stages III–IV periodontitis. Specifically, the median aMMP‐8 value for this group was 50 ng/mL (interquartile range: 22–86 ng/mL), and the aMMP‐8/NTP median was 2.1 (IQR: 1.0–3.5). In comparison, participants with stage II periodontitis had a median aMMP‐8 value of 30 ng/mL (IQR: 10–51 ng/mL) and an aMMP‐8/NTP median of 1.2 (IQR: 0.4–1.8). Those in the health/gingivitis/stage I periodontitis group had a median aMMP‐8 value of 11 ng/mL (IQR: 10–26 ng/mL) and an aMMP‐8/NTP median of 0.4 (IQR: 0.4–1.0). All these aMMP‐8 results exhibited significant associations with periodontitis.

Response frequencies for the modified 13‐item questionnaire are also displayed in Table 2. No “Refused” answers were recorded, while “Don't know” responses varied from 0.0% to 36.4% across the questionnaire. To calculate the OR in the univariable logistic regression analysis, “No/Never” and “Don't know” answers were combined for questions Q1, Q3a, Q3b, Q4, Q5, Q6, Q7, Q8, Q9, Q11, Q12, and Q13. For question Q2, the responses “Excellent,” “Very good,” and “Good” were merged. For question Q10, the responses “Very often,” “Fairly often,” and “Occasionally” were combined. Because of the relatively high proportion of “Don't know” responses to Q1 (30.0%), we further compared population characteristics of periodontal status, age, and socioeconomic factors among individuals with different Q1 answers (Figure S2 in the online *Journal of Periodontology*). No significant differences were observed in socioeconomic indicators (education and family income) across groups. However, individuals who selected “Don't know” exhibited age and periodontal status distributions more similar to those who answered “No”, both groups being generally younger and presenting a higher proportion of health/gingivitis/stage I periodontitis compared with those who answered “Yes.” The univariable logistic regression analysis indicated a significant association between the responses to questions Q1, Q2, Q3a, Q3b, Q4, Q5, Q6, Q7, Q10, Q11, Q12, and Q13 and the presence of periodontitis. Notably, the ORs were generally higher when predicting stages III–IV periodontitis compared with health, gingivitis, or stages I‐II periodontitis, suggesting that these questions have a stronger correlation with more advanced stages of periodontitis.

### 3.2 Comparison of diagnostic performance between models based on self‐test and quantitative reading aMMP‐8 results

**FIGURE 2:**
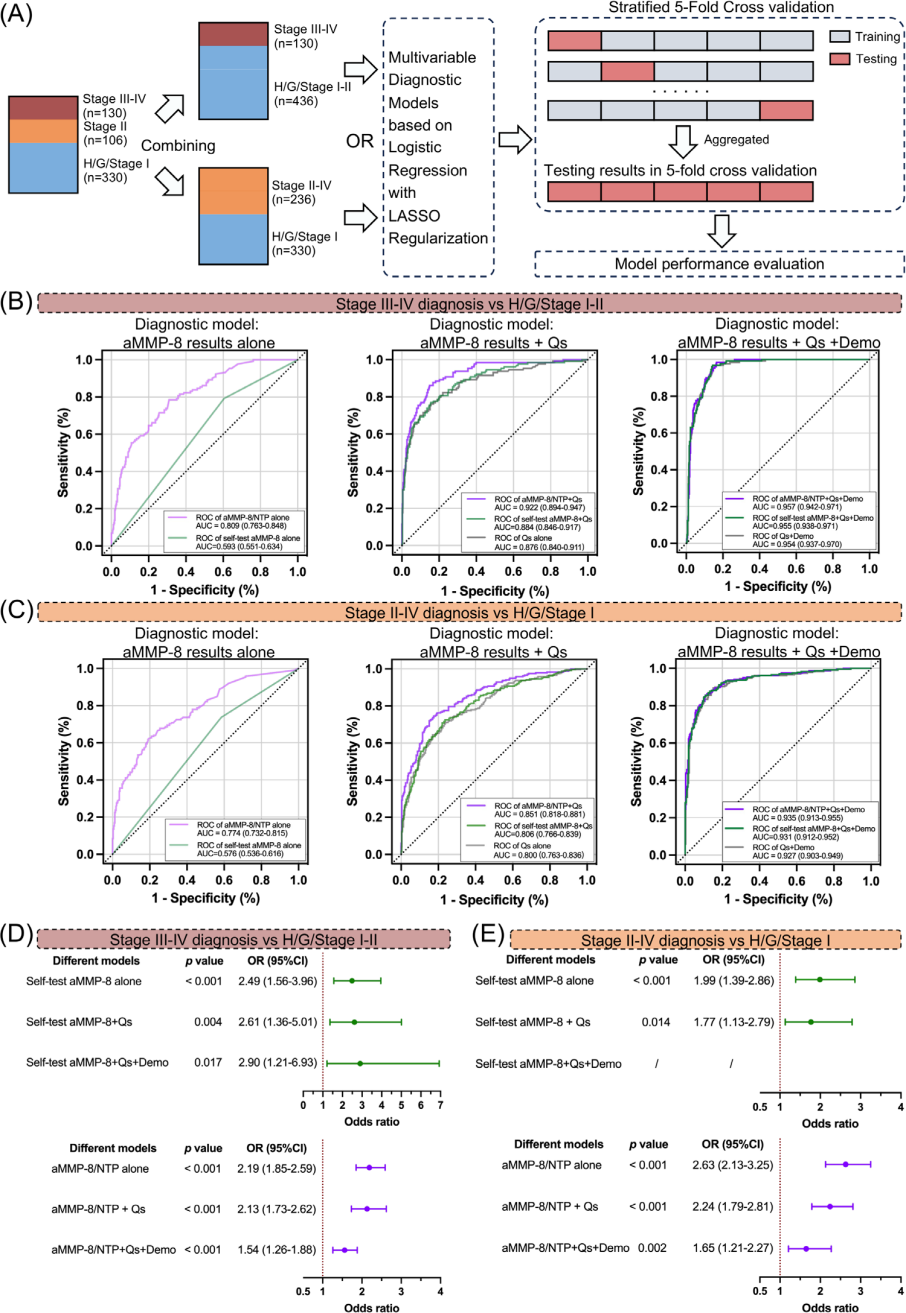
Diagnostic performance of univariable and multivariable models for different periodontal status. Diagnostic performance of univariable and multivariable models for different periodontal status, based on LASSO regularized logistic regression. (A) Schematic overview of the cross‐validation workflow used for multivariable model training and testing across different periodontal statuses; (B) ROC curves illustrating the diagnostic performance of various models based on the self‐tested and quantitative reading aMMP‐8 results, in combination with questionnaire and demographic predictors, for identifying stages III–IV periodontitis in the testing dataset; (C) ROC curves illustrating the diagnostic performance of various models based on the self‐test and quantitative reading aMMP‐8 results, in combination with questionnaire and demographic predictors, for identifying stages II–IV periodontitis in the testing dataset; (D) Logistic regression‐derived odds ratios for self‐test and quantitative‐reading aMMP‐8 results in different diagnostic models, indicating its contribution in predicting stages III–IV periodontitis; (E) Logistic regression‐derived odds ratios for self‐test and quantitative‐reading aMMP‐8 results in different diagnostic models, indicating its contribution in predicting stages II–IV periodontitis. Demo, demographic factors; LASSO, least absolute shrinkage and selection operator; Qs, questions in modified CDC/AAP questionnaire; ROC, receiver operating characteristics.

A series of diagnostic models were developed to evaluate the performance of self‐test versus quantitative readings of aMMP‐8 POCT results. Each aMMP‐8 result was analyzed alone, combined with the expanded CDC/AAP questionnaire, and with demographic factors. Predictor selection used LASSO regularization, and model training was conducted using stratified 5‐fold cross‐validation (Figures 2A).

The addition of questionnaires and demographic predictors significantly improved diagnostic performance (Figure 2B,C). Differences between self‐test and quantitative readings diminished in multivariable analyses, with both achieving high AUROC values. For stages II–IV periodontitis, the final models attained AUROC values of 0.931 for self‐test aMMP‐8 and 0.935 for quantitative readings. For stages III–IV periodontitis, AUROC values were 0.955 and 0.957, respectively.

While aMMP‐8 results contributed less to overall model performance, they remained statistically significant in most analyses. Self‐test aMMP‐8 showed stronger associations for stages III–IV prediction, while quantitative readings consistently demonstrated lower odds ratios (Figure 2D,E).

Summary performance metrics are presented in Tables 3 and S2 (online *Journal of Periodontology*). Univariate models exhibited low to moderate performance; however, multivariable models, particularly those incorporating questionnaires and demographic factors, showed improved performance. For stages III–IV, the self‐test model reached a sensitivity and specificity of 0.900 and 0.878, while the quantitative models achieved 0.908 and 0.878. For stages II–IV, the self‐reported model's sensitivity and specificity was 0.847 and 0.894, and the quantitative model was 0.831 and 0.894, respectively.

**TABLE 3:** Diagnostic performance of logistic regression models incorporating different combinations based on self‐reported aMMP‐8 results, questionnaire responses, and demographics for predicting periodontal status.

	Stages III–IV versus H/G/Stages I–II			Stages II–IV versus H/G/Stage I		
Selected predictors in models	Self‐reported aMMP‐8	Self‐reported aMMP‐8 + questionnaire	Self‐reported aMMP‐8 + questionnaire + demographics	Self‐reported aMMP‐8	Self‐reported aMMP‐8 + questionnaire	Self‐reported aMMP‐8 + questionnaire + demographics
**Odds ratio**						
Self‐reported aMMP‐8	2.49 (1.56‐3.96)^***^	2.61 (1.36–5.01)^**^	2.90 (1.21–6.93)^*^	1.99 (1.39‐2.86)^***^	1.77 (1.13–2.79)^*^	/
Q1: Gum disease	NA	2.01 (1.08–3.73)^*^	3.41 (1.40–8.32)^**^	NA	1.74 (1.09–2.78)^*^	2.51 (1.27–5.00)^**^
Q2: Rating of gum/teeth health		3.21 (1.23–8.40)^*^	/		4.13 (2.34–7.29)^***^	/
Q3a: Supragingival cleaning		/	/		1.87 (1.17–2.99)^**^	2.34 (1.12–4.92)^*^
Q4: Loose teeth		6.01 (3.43–10.53)^***^	5.60 (2.56–12.25)^***^		3.14 (1.86–5.32)^***^	/
Q5: Professionally diagnosed bone loss		3.54 (1.93–6.48)^***^	4.49 (1.90–10.61)^***^		2.54 (1.47–4.39)^***^	2.56 (1.21–5.42)^*^
Q6: Tooth appearance		/	/		/	/
Q8: Use of mouth rinse		/	/		/	2.19 (1.10–4.34)^*^
Q9: Bleeding on brushing		/	/		0.50 (0.28–0.88)^*^	/
Q10: Frequency of bleeding on brushing		/	/		/	2.98 (1.43–6.19)^**^
Q13: Food type changes		3.76 (1.70–8.31)^**^	3.96 (1.32–11.83)^*^		/	/
Age	NA	NA	1.15 (1.11–1.19)^***^	NA	NA	1.17 (1.13–1.21)^***^
Smoking status			1.97 (1.23–3.15)^**^			1.73 (1.10–2.71)^*^
**AUROC (95% CI)**	0.593 (0.551–0.634)	0.884 (0.846–0.917)	0.955 (0.938–0.971)	0.576 (0.536–0.616)	0.806 (0.766–0.839)	0.931 (0.912–0.952)
**Sensitivity (95% CI)**	0.792 (0.721–0.862)	0.715 (0.636–0.787)	0.900 (0.845–0.950)	0.737 (0.679–0.794)	0.665 (0.607–0.723)	0.847 (0.802–0.889)
**Specificity (95% CI)**	0.394 (0.351–0.441)	0.876 (0.844–0.907)	0.878 (0.846–0.908)	0.415 (0.364–0.466)	0.803 (0.761–0.842)	0.894 (0.859–0.927)
**Accuracy (95% CI)**	0.486 (0.445–0.527)	0.839 (0.809–0.873)	0.883 (0.857–0.908)	0.549 (0.512–0.590)	0.746 (0.712–0.781)	0.875 (0.846–0.903)

*Note*: Each model was constructed to predict either stages III‐IV or stages II‐IV periodontitis; multivariable models were regularized by LASSO. Performance metrics include odds ratio for selected predictors, AUROC, sensitivity, specificity and accuracy with 95% CI.The reference category for self‐reported aMMP‐8 is negative self‐reports; the reference category for Q1, Q3a, Q4, Q5, Q6, Q8, Q9, Q13 is the combination of “no” + “don't know” answers; the reference category for Q2 is the combination of answers “excellent” + “very good” + “good”; the reference category for Q10 is the combination of answers “very often” + “fairly often” + “occasionally”.

Abbreviations: 95% CI, confidence interval of 95%; aMMP‐8: activated matrix metalloproteinase‐8; AUROC: area under the receiver operating characteristic curve; LASSO: least absolute shrinkage and selection operator; NA: not applicable.

*** *p* < 0.001.

** *p* < 0.01.

* *p* < 0.05.

### 3.3 Age distribution of misclassified cases

**FIGURE 3:**
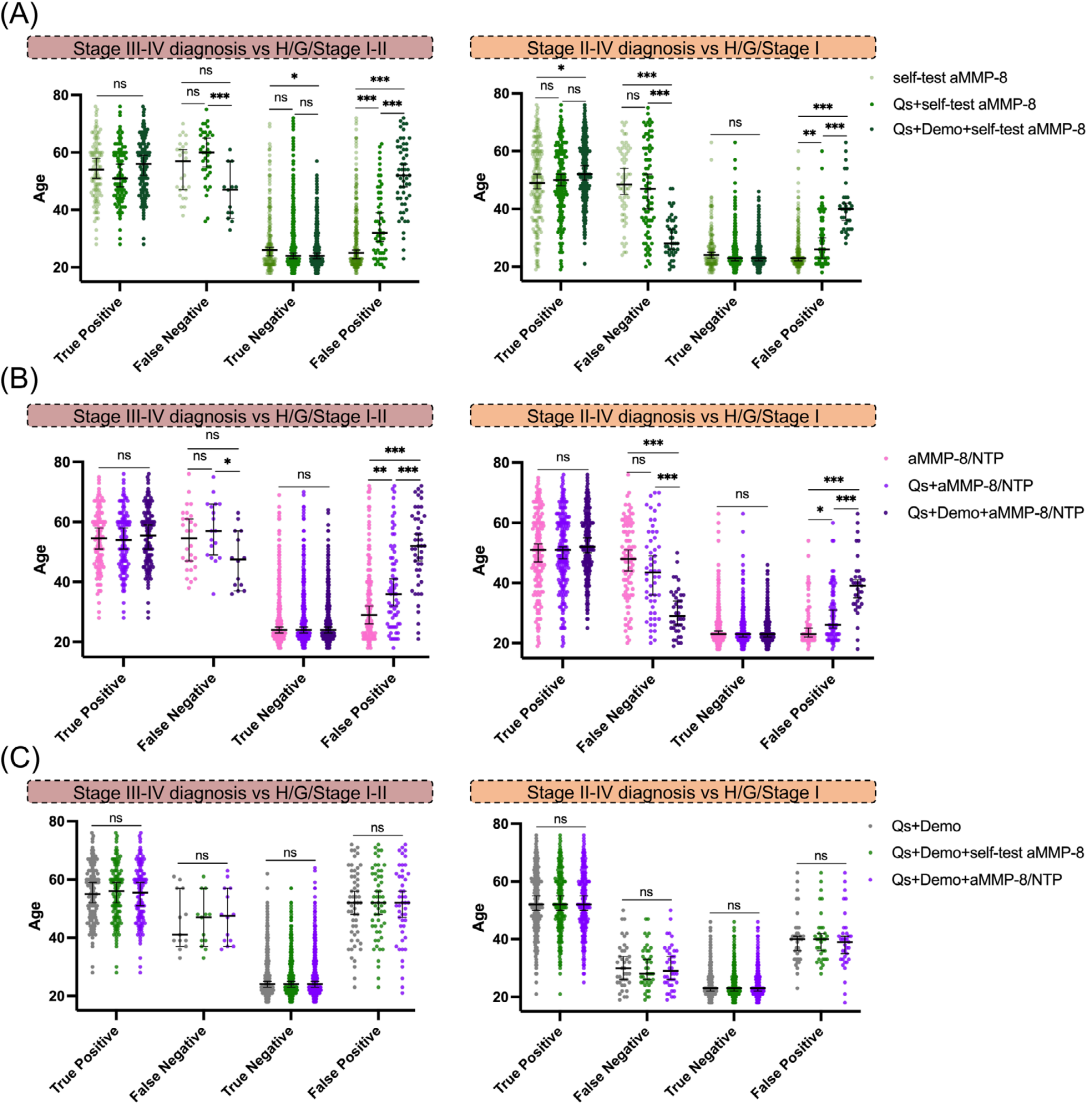
Misclassification analysis across age. The figure shows model misclassification across age distributions. (A) Age distribution of true positive, false negative, true negative and false positive cases of models based on self‐tested aMMP‐8, for distinguishing stages III–IV and stages II–IV periodontitis, respectively. (B) Age distribution of true positive, false negative, true negative and false positive cases under models based on quantitative reading aMMP‐8 (aMMP‐8/NTP), for distinguishing stages III–IV and stages II–IV periodontitis. (C) Age distribution of true positive, false negative, true negative, and false positive cases under models based on questionnaire and demographic variables, with or without inclusion of aMMP‐8 test results, for distinguishing stages III–IV and stages II–IV periodontitis. Kruskal–Wallis tests were conducted to compare age distributions across diagnostic models for each prediction category (true positive, false negative, true negative, or false positive). Post‐hoc pairwise comparisons between models were performed using Dunn test. ns: not statistically significant; * *p* < 0.05; ** *p* < 0.01; *** *p* < 0.001.

Given the high feature importance of age in the prediction models, the age distribution of true positive, false negative, true negative, and false positive cases across different diagnostic models was analyzed (Figure 3). In stages III–IV and stages II–IV periodontitis prediction (Figure 3A,B), adding demographic factors to models based on aMMP‐8 and questionnaire resulted in a significant shift: false negatives tended to be younger, while false positives were older. Despite improving overall model performance, this pattern showed the limitation of demographic predictors, especially age, which may hinder the accurate classification of atypical cases such as young individuals with disease or older individuals without.

As shown in Figure 3C, incorporating either self‐test or quantitative reading aMMP‐8 results into the full multivariable models with questionnaire and demographic factors did not significantly alter these age‐related misclassification trends. This observation suggests that current aMMP‐8 testing cannot resolve the inherent demographic bias in periodontitis prediction.

## 4 DISCUSSION

Advanced periodontitis can significantly impact both individual quality of life and public health costs, making effective screening tools urgently needed. Although symptoms become more apparent as the disease progresses, many individuals remain unaware of their condition.^45^ Developing accessible, validated, and cost‐effective multi‐modal screening tools is therefore crucial but remains challenging. This study confirms the high accuracy measures observed with models combining multiple nonclinical features of periodontitis^25^ and extends them to a group with periodontal health status determined by radiographic assessment. This study also reveals the limitations of multivariable diagnostic models incorporating aMMP‐8, questionnaires, and demographic information for self‐detection.

The aMMP‐8 test used has low sensitivity, and the high rates of false negatives make it unsuitable for use as a standalone diagnostic tool.^37^ While questionnaires show significant potential, carefully analyzing the important questions within the multivariable models across different geographical locations indicates that using a single algorithm may be ineffective, as different questions prove useful in different populations.^26^, ^29^, ^34^ Additionally, introducing demographic factors, particularly age, into the model can introduce bias in the distribution of missed predictions. A critical assessment of these models highlights their limitations: diagnostic odds ratios do not stabilize above generally accepted values, and confusion matrix analyses demonstrate age‐dependent performance. This results in false negatives among younger subjects and false positives in older ones when demographic information is included.

Previous studies have investigated various clinical scenarios for quantitative aMMP‐8 POCT, focusing primarily on differences in biomarker levels and its diagnostic utility in periodontitis and peri‐implantitis.^46^, ^47^ A recently published meta‐analysis indicated a sensitivity of 0.59 (95% CI: 0.42–0.75), specificity of 0.82 (95% CI: 0.68–0.93), and an AUROC of 0.77 (95% CI: 0.74–0.81) for quantitative aMMP‐8 in detecting periodontitis.^37^ These results are consistent with the performance observed in our univariable quantitative aMMP‐8 model for stages II–IV of periodontitis. As anticipated, the self‐test aMMP‐8 alone showed poor diagnostic performance in the univariable analysis, likely due to its binary nature. While a positive self‐test result may suggest disease presence, the lack of a continuous scale limits its ability to identify borderline or early‐stage cases, thus reducing its diagnostic performance.

Furthermore, our findings, along with those from other studies, indicate that quantitative aMMP‐8 results alone are inadequate for periodontitis screening. Researchers have explored combining quantitative aMMP‐8 data with self‐reported questionnaires (e.g., the CDC/AAP questionnaire) and/or demographic risk factors^24^, ^29^ to enhance diagnostic accuracy. Our findings also support this combined approach: in both stages II–IV and stages III–IV predictions, integrating the modified CDC/AAP questionnaire significantly improved model performance, and further enhancements were observed when demographic factors were included. These multivariable models achieved high AUROC values, sensitivity, and specificity, showcasing their potential utility in periodontitis detection.

A crucial point about our results must be emphasized. Despite the relevance of incorporating aMMP‐8 into our models, this biomarker did not significantly enhance the performance of a model based solely on questionnaire and demographic data. This finding deserves clear understanding. Previous work has highlighted the cultural and geographical limitations of using the CDC/AAP questionnaire.^45^ A logistic model must be tailored to account for specific population characteristics, such as access to care, previous diagnoses of bone loss, prior treatments, or particular oral hygiene practices. In addition, the present population shows a high educational status, which may bias the performance of the questionnaires. However, in the analysis focusing on population characteristics by responses to CDC/AAP questionnaire item Q1, the three groups (“Yes”, “No”, and “Don't know”) showed broadly similar distributions of socioeconomic factors (Figure S2). Nevertheless, nearly 30% of individuals answering “No” or “Don't know” were diagnosed with stages II–IV periodontitis, highlighting a critical lack of self‐awareness regarding periodontal health. These findings underscore the limitations of relying solely on self‐reported questionnaires for periodontal disease screening and emphasize the need to strengthen public education and awareness to improve recognition of periodontal disease. Although not clearly demonstrated in our findings, integrating objective biomarkers could theoretically help mitigate issues related to self‐perception and enhance the cross‐cultural validity of screening strategies.

Another important consideration is the significant contribution of demographics to the predictive model. In our analysis, “age” consistently accounted for a more substantial share of predictive power, while aMMP‐8 results provided only marginal improvements, aligning with our earlier findings.^24^ However, even the strong predictor “age” has notable limitations, as it enhances overall model performance but reduces the ability to accurately classify atypical subpopulations, such as younger individuals with periodontitis or older individuals without. This limitation underscores the challenges of relying on demographic factors to determine periodontal health status. Biomarkers can offer direct biological evidence independent of variables like age, geography, or culture, but in this study, the aMMP‐8 test did not fulfill this role. Its limited diagnostic contribution did not assist in resolving misclassifications in these demographic edge cases. Our recent work highlighted the limitations of aMMP‐8 oral rinses in detecting localized periodontitis.^48^ This study expands the existing evidence to a group of subjects whose periodontal status has been defined through radiographic analysis, further emphasizing the need for more sensitive, age‐independent diagnostic tools, which might include new biomarkers, refined testing approaches, or alternative diagnostic methods.

Another key finding in this study is that diagnostic performance was consistently higher across all models predicting stages III–IV periodontitis than stages II–IV. This observation aligns with previous findings on aMMP‐8 expression in relation to periodontitis severity: increased collagen degradation and inflammatory activity in more advanced disease led to more pronounced changes in aMMP‐8 expression.^49^ From a biological perspective, this explains why more severe disease is easier to detect. Clinically, advanced periodontitis is characterized by pronounced attachment loss, bone destruction, and gingival inflammation, leading to overt symptoms such as tooth mobility, aesthetic concerns, and masticatory dysfunction.^10^, ^39^, ^50^ These progressive changes, from biological alterations to clinical manifestations to perceptible symptoms, enhance the detectability of advanced disease, making it inherently more distinguishable than early‐stage periodontitis. In contrast, stage I periodontitis often presents with subtle or minimal clinical signs, which limits the accuracy of both current clinical probing and nonclinical diagnostic tools. Nevertheless, early detection of stage I periodontitis is critically important, as it represents the onset of the disease and is frequently overlooked or misjudged in clinical practice. Therefore, developing reliable approaches for the timely and accurate diagnosis of early‐stage periodontitis remains an urgent and essential goal for future research.

Several limitations should be acknowledged. First, due to data availability, radiographic bone loss was used as the reference diagnosis. This approach restricted the classification of periodontal health and gingivitis and allowed assessment of periodontitis stage only in terms of severity, without accounting for complexity. Accordingly, periodontal health and gingivitis were combined with stage I periodontitis, while stage III and stage IV periodontitis were analyzed together, as the available data did not permit reliable differentiation between them. Second, radiographic bone loss may also result from factors unrelated to periodontal disease, such as previous tooth extraction or tooth malposition. The absence of corresponding clinical examination data in this study limited our ability to distinguish these causes and prevented correction for non‐periodontal bone loss. Third, information on smoking status and medical history of systemic diseases was self‐reported by participants, which may have introduced reporting bias. Despite these limitations, this study has several strengths: (i) it evaluated the diagnostic potential of self‐test aMMP‐8 results and their multivariable model for periodontitis; (ii) it involved an appropriate sample size with sufficient statistical power; (iii) both the index tests and reference standards were assessed in a blind manner; and (iv) the study provided comprehensive data supporting its conclusions.

## 5 CONCLUSION

This study evaluated the diagnostic potential of self‐test aMMP‐8 results for periodontitis using a framework that combines the modified CDC/AAP questionnaire and demographic factors. Through LASSO‐regularized logistic regression and internal cross‐validation, we developed and preliminarily assessed diagnostic algorithms based on this biomarker. The multi‐modal models based on self‐test aMMP‐8 demonstrated diagnostic performance comparable to that based on quantitative aMMP‐8. These findings identified both the challenges of self‐awareness and the limited precision of current self‐test but also indicated the feasibility of a multi‐modal self‐reported screening tool for periodontitis that is scalable, low‐cost, and accessible in community and public health settings. Further development and validation in a more diverse population are needed to confirm its generalizability and effectiveness.

## AUTHOR CONTRIBUTIONS

Yu Xie and Xiaoyu Yu contributed equally to this article. Yu Xie and Xiaoyu Yu contributed to protocol development, data collection, analysis, and interpretation, and manuscript preparation. Mengning Bi and Harui Li contributed to experimental work. Yuan Li contributed to protocol development, data interpretation, and manuscript preparation. Maurizio S. Tonetti devised this study and contributed to protocol development, data interpretation, and manuscript preparation. All authors contributed to the manuscript revision, gave their final approval, and agreed to be accountable for all aspects of the work.

## CONFLICT OF INTEREST STATEMENT

Maurizio S. Tonetti received grant support and/or personal fees from Geistlich Pharma AG (Wolhusen, Switzerland), Straumann AG (Basel, Switzerland), and Nobel Biocare SA (Zurich, Switzerland), which are unrelated to the present work. The other authors declared no conflicts of interest.

## Supporting information



Supporting information

## Data Availability

The data that support the findings of this study are available for research collaboration from the corresponding author upon reasonable request.

## Footnotes

* PASS, 2023, NCSS LLC, Kaysville, Utah, USA

† PerioSafe PRO, Dentognostics GmbH, Jena, Germany

‡ ORALyzer, Dentognostics GmbH, Jena, Germany

§ Version 26.0, Statistical Package for the Social Sciences, IBM Corporation, Armonk, New York, USA

## References

[1] Jiao J , Jing W , Si Y , et al. The prevalence and severity of periodontal disease in Mainland China: data from the Fourth National Oral Health Survey (2015‐2016). J Clin Periodontol. 2021;48(2):168‐179. doi:10.1111/jcpe.13396 33103285

[2] Papapanou PN , Susin C . Periodontitis epidemiology: is periodontitis under‐recognized, over‐diagnosed, or both?. Periodontol 2000. 2017;75(1):45‐51. doi:10.1111/prd.12200 28758302

[3] Genco RJ , Sanz M . Clinical and public health implications of periodontal and systemic diseases: an overview. Periodontol 2000. 2020;83(1):7‐13. doi:10.1111/prd.12344 32385880

[4] Tonetti MS , D'Aiuto F , Nibali L , et al. Treatment of periodontitis and endothelial function. N Engl J Med. 2007;356(9):911‐920. doi:10.1056/NEJMoa063186 17329698

[5] Liu M , Liu B , Shen J , et al. Low energy intake and nutritional maladaptation in terminal stage IV periodontitis. J Clin Periodontol. 2024;51(9):1147‐1156. doi:10.1111/jcpe.14022 38807437

[6] Sabbah W , Gomaa N , Gireesh A . Stress, allostatic load, and periodontal diseases. Periodontol 2000. 2018;78(1):154‐161. doi:10.1111/prd.12238 30198126

[7] Varela‐Centelles P , Diz‐Iglesias P , Estany‐Gestal A , Seoane‐Romero JM , Bugarín‐González R , Seoane J . Periodontitis awareness amongst the general public: a critical systematic review to identify gaps of knowledge. J Periodontol. 2016;87(4):403‐415. doi:10.1902/jop.2015.150458 26545044

[8] Sharma P , Yonel Z , Busby M , Chapple IL , Dietrich T . Association between periodontal health status and patient‐reported outcomes in patients managed in a non‐specialist, general dental practice. J Clin Periodontol. 2018;45(12):1440‐1447. doi:10.1111/jcpe.13022 30341963

[9] Levin L . Aggressive periodontitis: the silent tooth killer. Alpha Omegan. 2011;104(3/4):74. 22686102

[10] Buset SL , Walter C , Friedmann A , Weiger R , Borgnakke WS , Zitzmann NU . Are periodontal diseases really silent? A systematic review of their effect on quality of life. J Clin Periodontol. 2016;43(4):333‐344. doi:10.1111/jcpe.12517 26810308

[11] Pattamatta M , Chapple I , Listl S . The value‐for money of preventing and managing periodontitis: opportunities and challenges. Periodontol 2000. 2024. Published online September 14, 2024. doi:10.1111/prd.12569 PMC12842866 38745388

[12] Wu L , Zhang SQ , Zhao L , Ren ZH , Hu CY . Global, regional, and national burden of periodontitis from 1990 to 2019: results from the Global Burden of Disease study 2019. J Periodontol. 2022;93(10):1445‐1454. doi:10.1002/JPER.21-0469 35305266

[13] Holtfreter B , Kuhr K , Borof K , et al. ACES: a new framework for the application of the 2018 periodontal status classification scheme to epidemiological survey data. J Clin Periodontol. 2024;51(5):512‐521. doi:10.1111/jcpe.13965 38385950

[14] Watts T . Constant force probing with and without a stent in untreated periodontal disease: the clinical reproducibility problem and possible sources of error. J Clin Periodontol. 1987;14(7):407‐411. doi:10.1111/j.1600-051x.1987.tb01545.x 3476518

[15] Salvi GE , Roccuzzo A , Imber JC , Stähli A , Klinge B , Lang NP . Clinical periodontal diagnosis. Periodontol. 2000;2023:1‐19. doi:10.1111/prd.12487 37452444

[16] Benigeri M , Brodeur JM , Payette M , Charbonneau A , Ismaïl AI . Community periodontal index of treatment needs and prevalence of periodontal conditions. J Clin Periodontol. 2000;27(5):308‐312. doi:10.1034/j.1600-051x.2000.027005308.x 10847533

[17] Cutress T , Ainamo J , Sardo‐Infirri J . The community periodontal index of treatment needs (CPITN) procedure for population groups and individuals. Int Dent J. 1987;37(4):222‐233. doi:10.1111/j.1875-595X.1987.tb00465.x 3481626

[18] Lang NP , Hill RW . Radiographs in periodonties. J Clin Periodontol. 1977;4(1):16‐28. doi:10.1111/j.1600-051x.1977.tb01879.x 321483

[19] Tonetti MS , Sanz M . Implementation of the new classification of periodontal diseases: decision‐making algorithms for clinical practice and education. J Clin Periodontol. 2019;46(4):398‐405. doi:10.1111/jcpe.13104 30883878

[20] del Rosario M , Mikhail M . The world health organization global initiative on radiation safety in healthcare settings. In: Vetter RJS , Magdalena S , eds. Radiation Protection in Medical Imaging and Radiation Oncology. CRC Press; 2015:325‐340. doi:10.1201/b19063

[21] Malone J , del Rosario Perez M , Friberg EG , et al. Justification of CT for individual health assessment of asymptomatic persons: a World Health Organization consultation. J Am Coll Radiol. 2016;13(12):1447‐1457.e1. doi:10.1016/j.jacr.2016.07.020 27916111 PMC5357768

[22] Andrade FB , Antunes JLF , Andrade FCD , Lima‐Costa M , Macinko J . Education‐related inequalities in dental services use among older adults in 23 countries. J Dent Res. 2020;99(12):1341‐1347. doi:10.1177/0022034520935854 32623932 PMC7580169

[23] Manski R , Rohde F , Ricks T , Chalmers NI . Trends in the Number and Percentage of the Population with any Dental or Medical Visits, 2019. In: Statistical Brief (Medical Expenditure Panel Survey (US)). Agency for Healthcare Research and Quality (US) ; Rockville, MD; 2023. 37616435

[24] Deng K , Zonta F , Yang H , Pelekos G , Tonetti MS . Development of a machine learning multiclass screening tool for periodontal health status based on non‐clinical parameters and salivary biomarkers. J Clin Periodontol. 2024;51(12):1547‐1560. doi:10.1111/jcpe.13856 37697491 PMC11651717

[25] Carra MC , Gueguen A , Thomas F , et al. Self‐report assessment of severe periodontitis: periodontal screening score development. J Clin Periodontol. 2018;45(7):818‐831. doi:10.1111/jcpe.12899 29611224

[26] Arias‐Bujanda N , Regueira‐Iglesias A , Balsa‐Castro C , Nibali L , Donos N , Tomás I . Accuracy of single molecular biomarkers in saliva for the diagnosis of periodontitis: a systematic review and meta‐analysis. J Clin Periodontol. 2020;47(1):2‐18. doi:10.1111/jcpe.13202 31560804

[27] Grant MM , Taylor JJ , Jaedicke K , et al. Discovery, validation, and diagnostic ability of multiple protein‐based biomarkers in saliva and gingival crevicular fluid to distinguish between health and periodontal diseases. J Clin Periodontol. 2022;49(7):622‐632. doi:10.1111/jcpe.13630 35451104 PMC9324935

[28] Eke P , Dye B , Wei L , et al. Self‐reported measures for surveillance of periodontitis. J Dent Res. 2013;92(11):1041‐1047. doi:10.1177/0022034513505621 24065636

[29] Deng K , Pelekos G , Jin L , Tonetti MS . Diagnostic accuracy of a point‐of‐care aMMP‐8 test in the discrimination of periodontal health and disease. J Clin Periodontol. 2021;48(8):1051‐1065. doi:10.1111/jcpe.13485 33998040 PMC8362205

[30] Goulão B , MacLennan GS , Ramsay CR . Have you had bleeding from your gums? Self‐report to identify gingival inflammation (the SING diagnostic accuracy and diagnostic model development study). J Clin Periodontol. 2021;48(7):919‐928. doi:10.1111/jcpe.13455 33751629

[31] Eke PI , Genco RJ . CDC periodontal disease surveillance project: background, objectives, and progress report. J Periodontol. 2007;78(suppl 7):1366‐1371. doi:10.1902/jop.2007.070134 10.1902/jop.2007.070134 17610396

[32] Eke PI , Thornton‐Evans G , Dye B , Genco R . Advances in surveillance of periodontitis: the Centers for Disease Control and Prevention periodontal disease surveillance project. J Periodontol. 2012;83(11):1337‐1342. doi:10.1902/jop.2012.110676 22324489 PMC6004792

[33] Deng K , Pelekos G , Jin L , Tonetti MS . Diagnostic accuracy of self‐reported measures of periodontal disease: a clinical validation study using the 2017 case definitions. J Clin Periodontol. 2021;48(8):1037‐1050. doi:10.1111/jcpe.13484 33998009

[34] Wei S , Lin T , Sáenz‐Ravello G , et al. Diagnostic accuracy of salivary active matrix metalloproteinase (aMMP)‐8 point‐of‐care test for detecting periodontitis in adults: a systematic review and meta‐analysis. J Clin Periodontol. 2024;51(8):1093‐1108. doi:10.1111/jcpe.14000 38763168

[35] Deng K , Wei S , Xu M , Shi J , Lai H , Tonetti MS . Diagnostic accuracy of active matrix metalloproteinase‐8 point‐of‐care test for the discrimination of periodontal health status: comparison of saliva and oral rinse samples. J Periodontal Res. 2022;57(4):768‐779. doi:10.1111/jre.12999 35575900

[36] Cohen JF , Korevaar DA , Altman DG , et al. STARD 2015 guidelines for reporting diagnostic accuracy studies: explanation and elaboration. BMJ Open. 2016;6(11):e012799. doi:10.1136/bmjopen-2016-012799 PMC5128957 28137831

[37] Li Y , Kung JCK , Shi J , et al. Diagnostic accuracy of a point‐of‐care aMMP‐8 test for discriminating periodontal health status in adults: validation trials and updated meta‐analysis. J Clin Periodontol. 2025;52(4):510‐529. doi:10.1111/jcpe.14119 39806539 PMC11949621

[38] Laudisio A , Marzetti E , Pagano F , Bernabei R , Zuccalà G . Masticatory dysfunction is associated with worse functional ability: a population‐based study. J Clin Periodontol. 2010;37(2):113‐119. doi:10.1111/j.1600-051X.2009.01518.x 20041974

[39] Uy SN , Deng K , Fok CTC , Fok MR , Pelekos G , Tonetti MS . Food intake, masticatory function, tooth mobility, loss of posterior support, and diminished quality of life are associated with more advanced periodontitis stage diagnosis. J Clin Periodontol. 2022;49(3):240‐250. doi:10.1111/jcpe.13588 34935175

[40] Bassiouny M , Grant A . The accuracy of the Schei ruler: a laboratory investigation. J Periodontol. 1975;46(12):748‐752. doi:10.1902/jop.1975.46.12.748 1060755

[41] Papapanou PN , Sanz M , Buduneli N , et al. Periodontitis: consensus report of workgroup 2 of the 2017 world workshop on the classification of periodontal and peri‐implant diseases and conditions. J Periodontol. 2018;89:S173‐S182. doi:10.1002/JPER.17-0721 29926951

[42] Tonetti MS , Greenwell H , Kornman KS . Staging and grading of periodontitis: framework and proposal of a new classification and case definition. J Periodontol. 2018;89:S159‐S172. doi:10.1002/JPER.18-0006 29926952

[43] Nelson DE , Holtzman D , Bolen J , Stanwyck CA , Mack KA . Reliability and validity of measures from the Behavioral Risk Factor Surveillance System (BRFSS). Soz Praventivmed. 2001;46:S3‐42. doi:10.1007/BF01299946 11851091

[44] Swets JA . Measuring the accuracy of diagnostic systems. Science. 1988;240(4857):1285‐1293. doi:10.1126/science.3287615 3287615

[45] Bi M , Xie Y , Yu X , Li H , Li Y , Tonetti MS . Diagnostic accuracy of self‐reported questionnaires for detecting periodontitis across multiple cultures and geographic locations: a systematic review and meta‐analysis. J Clin Periodontol. 2025. Published online August 8, 2025. doi:10.1111/jcpe.70002 PMC12531362 40781755

[46] Lähteenmäki H , Umeizudike KA , Heikkinen AM , et al. aMMP‐8 point‐of‐care/chairside oral fluid technology as a rapid, non‐invasive tool for periodontitis and peri‐implantitis screening in a medical care setting. Diagnostics (Basel). 2020;10(8):562. doi:10.3390/diagnostics10080562 32764436 PMC7460514

[47] Räisänen IT , Aji N , Sakellari D , et al. Active matrix metalloproteinase‐8 (aMMP‐8) versus total MMP‐8 in periodontal and peri‐implant disease point‐of‐care diagnostics. Biomedicines. 2023;11(11):2885. doi:10.3390/biomedicines11112885 38001886 PMC10669684

[48] Bi M , Xie Y , Yu X , et al. Clinical features associated with periodontal case misclassification by an active matrix metalloproteinase‐8 coint‐of‐care oral rinse test. J Clin Periodontol. 2025;52(9):1276‐1287. doi:10.1111/jcpe.14189 40462486 PMC12377944

[49] He W , You M , Wan W , Xu F , Li F , Li A . Point‐of‐care periodontitis testing: biomarkers, current technologies, and perspectives. Trends Biotechnol. 2018;36(11):1127‐1144. doi:10.1016/j.tibtech.2018.05.013 30041883

[50] Deng K , Uy SN , Fok C , Fok MR , Pelekos G , Tonetti MS . Assessment of masticatory function in the differential diagnosis of Stage IV periodontitis: a pilot diagnostic accuracy study. J Periodontol. 2022;93(6):803‐813. doi:10.1002/JPER.21-0660 35239983

