# Supplementary material for: aMMP‐8 self‐testing and self‐administered questionnaires for periodontitis screening: A diagnostic trial in a Chinese population
Source: J Periodontol. 2025 Dec 10;97(6):1300–16. doi: 10.1002/jper.70032 (PMC13350311; doi:10.1002/jper.70032)
Supplement: Supplementary file 1 — Supporting information [file JPER-97-1300-s001.docx]

**Appendix**

**aMMP-8 Self-Testing and Self-Administered Questionnaires for Periodontitis Screening: a Diagnostic Trial in a Chinese Population**

Yu Xie^1,2*^, Xiaoyu Yu^1,2*^, Mengning Bi^1,2^, Hairui Li^1,2^, Yuan Li^1,2,3^, Maurizio S. Tonetti^1,2,4^

*These authors contributed equally to this article

^1^ Shanghai PerioImplant Innovation Center, Institute for Oral, Craniofacial and Sensory Research, Ninth People's Hospital, Shanghai Jiao Tong University School of Medicine, Shanghai, China

^2^ National Clinical Research Center of Oral Diseases and National Center of Stomatology, College of Stomatology, Shanghai Jiao Tong University School of Medicine, Shanghai, China

^3^ Department of Oral Implantology, Ninth People's Hospital, Shanghai, China

^4^ European Research Group on Periodontology (ERGOPerio), Genova, Italy

**Correspondence**: Maurizio S. Tonetti, Perio-Implant Innovation Center, 4F Building 1, 115 Jinzun Road, Pudong Research Campus, Shanghai Jiao Tong University School of Medicine, 200115, Shanghai, China.

email: [maurizio.tonetti@ergoperio.eu](mailto:maurizio.tonetti@ergoperio.eu)

TABLE S1 The original and Chinese translations of the questions from the expanded CDC/AAP questionnaire.

|  | **Self-report Questions (translations in English/Chinese)** | | |
| --- | --- | --- | --- |
| 1. | Do you think you might have gum disease? | | |
|  | ☐Yes | ☐No | ☐Don't know |
|  | 你认为你有牙周病吗？ | |  |
|  | ☐有 | ☐没有 | ☐不知道 |
| 2. | Overall, how would you rate the health of your teeth and gum? | | |
|  | ☐Excellent |  |  |
|  | ☐Very Good | |  |
|  | ☐Good |  |  |
|  | ☐Fair |  |  |
|  | ☐Poor |  |  |
|  | ☐Don't know | |  |
|  | 总体而言，你会如何评价你牙齿和牙龈（牙肉、牙床）的健康状况？ | | |
|  | ☐绝佳 |  |  |
|  | ☐非常好 |  |  |
|  | ☐好 |  |  |
|  | ☐一般 |  |  |
|  | ☐差 |  |  |
|  | ☐不知道 |  |  |
| 3a. | Have you ever had treatment for gum disease, such as supragingival scaling? | | |
|  | ☐Yes | ☐No | ☐Don't know |
|  | 你有洗过牙吗？ | |  |
|  | ☐有 | ☐没有 | ☐不知道 |
| 3b. | Have you ever had treatment for gum disease, such as subgingival scaling and root planning, sometimes called “deep cleaning”? | | |
|  | ☐Yes | ☐No | ☐Don't know |
|  | 你有接受过任何牙周病相关的治疗吗？比如牙根刮治，也称为深层洗牙/清洁。 | | |
|  | ☐有 | ☐没有 | ☐不知道 |
| 4. | Have you ever had any teeth become loose on their own, without an injury? | | |
|  | ☐Yes | ☐No | ☐Don't know |
|  | 你的牙齿在没有任何创伤或意外下，曾经有变得松动吗？ | | |
|  | ☐有 | ☐没有 | ☐不知道 |
| 5. | Have you ever been told by a dental professional that you lost bone around your teeth? | | |
|  | ☐Yes | ☐No | ☐Don't know |
|  | 过往曾经有口腔医师/牙医告知你，牙齿周围的骨头有吸收或萎缩吗？ | | |
|  | ☐有 | ☐没有 | ☐不知道 |
| 6. | During the past 3 months, have you noticed a tooth that doesn’t look right? (n/%) | | |
|  | ☐Yes | ☐No | ☐Don't know |
|  | 在过去的三个月中，你有发觉牙齿有任何异样吗？ | | |
|  | ☐有 | ☐没有 | ☐不知道 |
| 7. | Aside from brushing your teeth with a toothbrush, in the last 7 days, how many times did you use dental floss or any other device to clean between your teeth? | | |
|  |  | Number |  |
|  | 除了用牙刷刷牙外，在过去七天内，你用了多少次牙线或其他清洁工具去清洁牙缝？ | | |
|  |  | 次 |  |
| 8. | Aside from brushing your teeth with a toothbrush, in the last 7 days, how many times did you use mouthwash or other dental rinse product that you use to treat dental disease or dental problems? | | |
|  |  | Number |  |
|  | 除了用牙刷刷牙外，在过去七天内，你用了多少次漱口水或其他口腔漱口产品去改善口腔问题？ | | |
|  |  | 次 |  |
| 9. | Have you ever had bleeding gums when brushing your teeth? | | |
|  | ☐Yes | ☐No | ☐Don't know |
|  | 你是否（或曾经）有过刷牙出血？ | | |
|  | ☐有 | ☐没有 | ☐不知道 |
| 10. | How often did you have bleeding gums when brushing your teeth? | | |
|  | ☐Very often | |  |
|  | ☐Fairly often | |  |
|  | ☐Occasionally | |  |
|  | ☐Seldom |  |  |
|  | ☐Never |  |  |
|  | 你是否经常出现刷牙出血？ | | |
|  | ☐很经常 |  |  |
|  | ☐经常 |  |  |
|  | ☐有时 |  |  |
|  | ☐很少 |  |  |
|  | ☐从未 |  |  |
| 11. | Do you experience difficulty chewing? | | |
|  | ☐Yes | ☐No | ☐Don't know |
|  | 你是否存在咀嚼困难/吃力？ | | |
|  | ☐有 | 没有 | 不知道 |
| 12. | Has the amount of food you usually eat decreased in the last year because of chewing problems? | | |
|  | ☐Yes | ☐No | ☐Don't know |
|  | 你过去一年的食量是否因为咀嚼困难/吃力而有所减少？ | | |
|  | ☐有 | ☐没有 | ☐不知道 |
| 13. | Did you need to change the type of food you eat because of chewing problems? | | |
|  | ☐Yes | ☐No | ☐Don't know |
|  | 由于咀嚼困难/吃力，你是否需要调整或改变饮食（所吃的食物）的类型？ | | |
|  | ☐有 | ☐没有 | ☐不知道 |

FIGURE S1. STARD Diagram


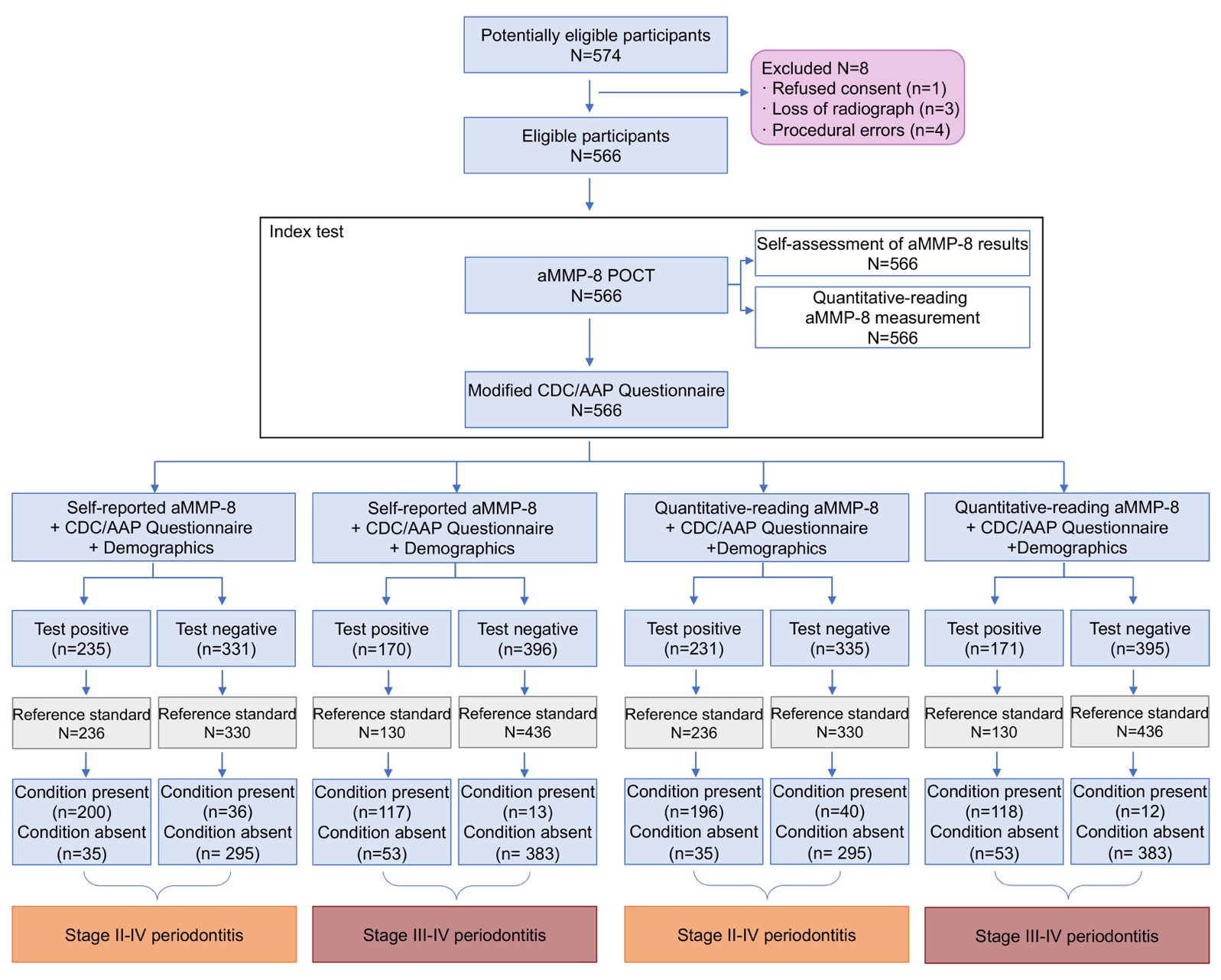


FIGURE S2. Population analysis of periodontal status, age and socioeconomic factors in relation to CDC/AAP questionnaire item Q1 on self-reported gum disease.


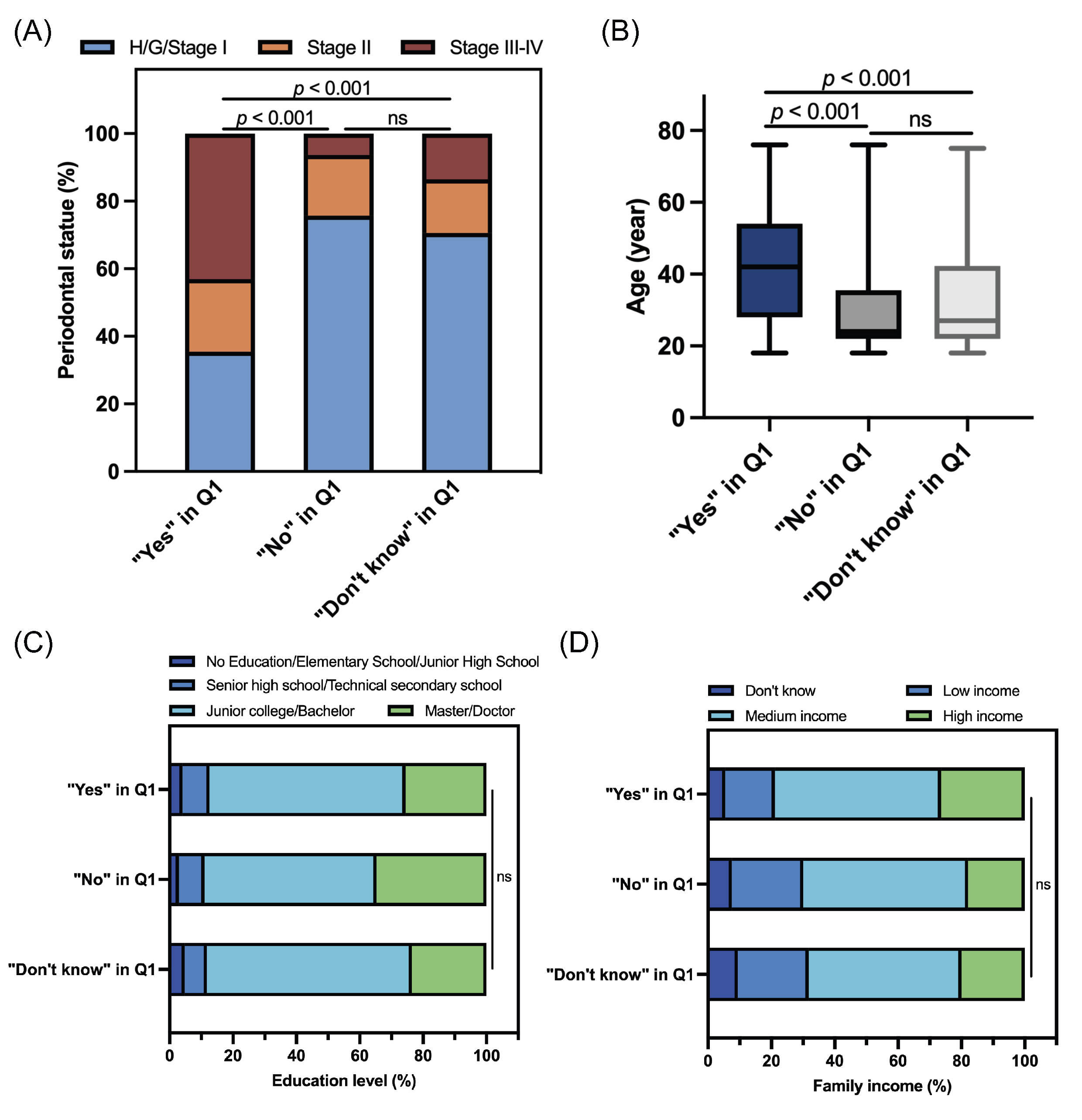


Population characteristics were compared among individuals who answered “Yes”, “No” or “Don’t know” to the question “Do you think you might have gum disease?” from the CDC/AAP questionnaire. (A) Distribution of periodontal status across groups, assessed using the Chi-square test. (B) Age distribution and group comparison, assessed using the Kruskal-Wallis test, with post-hoc pairwise comparisons performed using Dunn’s test. (C) Distribution of family income across groups, assessed using the Chi-square test. (D) Distribution of education levels across groups, assessed using the Chi-square test. Statistical analyses were performed both across all three groups and for post-hoc pairwise comparisons between each pair of groups. Statistical significance is indicated as *p* < 0.05; “ns” denotes no statistically significant difference.

TABLE S2 Diagnostic performance of logistic regression models incorporating different combinations based on quantitative reading aMMP-8 results, questionnaire responses, and demographics for predicting periodontal status. Each model was constructed to predict either stage III-IV or stage II-IV periodontitis, multivariable models were regularized by LASSO. Performance metrics include odds ratio for selected predictors, AUROC, sensitivity, specificity and accuracy with 95%CI.

| **Selected predictors**  **in models** | | **Stage III-IV vs H/G/Stage I-II** | | |  | **Stage II-IV vs H/G/Stage I** | | |
| --- | --- | --- | --- | --- | --- | --- | --- | --- |
|  |  | **Quantitative reading aMMP-8** | **Quantitative reading aMMP-8**  **+**  **Questionnaire** | **Quantitative reading aMMP-8**  **+**  **Questionnaire**  **+**  **Demographics** |  | **Quantitative reading aMMP-8** | **Quantitative reading aMMP-8**  **+**  **Questionnaire** | **Quantitative reading aMMP-8**  **+**  **Questionnaire**  **+**  **Demographics** |
| **Odds ratio** | |  |  |  |  |  |  |  |
|  | aMMP-8/NTP | 2.19 (1.85-2.59)^***^ | 2.13 (1.73-2.62)^***^ | 1.65 (1.27-2.16)^***^ |  | 2.63 (2.13-3.25)^***^ | 2.24 (1.79-2.81)^***^ | 1.65 (1.21-2.27)^**^ |
|  | Q1: Gum disease | NA | 2.22 (1.12-4.39)^*^ | 3.66 (1.47-9.13)^**^ |  | NA | 1.73 (1.05-2.86)^*^ | 2.61 (1.32-5.17)^**^ |
|  | Q2: Rating of gum/teeth health |  | 3.92 (1.28-11.98)^*^ | / |  |  | 4.06 (2.19-7.50)^***^ | / |
|  | Q3a: Supragingival cleaning |  | / | / |  |  | 1.81 (1.09-3.00)^*^ | 2.20 (1.05-4.62)^*^ |
|  | Q4: Loose teeth |  | 6.98 (3.68-13.26)^***^ | 6.22 (2.77-13.98)^***^ |  |  | 2.74 (1.55-4.85)^***^ | / |
|  | Q5: Professionally diagnosed bone loss |  | 3.94 (2.01-7.74)^***^ | 4.50 (1.88-10.80)^***^ |  |  | 2.69 (1.49-4.85)^**^ | 2.71 (1.27-5.77)^**^ |
|  | Q6: Tooth appearance |  | 0.50 (0.25-0.98)^*^ | / |  |  | / | / |
|  | Q8: Use of mouth rinse |  | / | / |  |  | / | 2.31 (1.16-4.61)^*^ |
|  | Q9: Bleeding on brushing |  | / | / |  |  | 0.50 (0.27-0.92)^*^ | / |
|  | Q10: Frequency of bleeding on brushing |  | / | / |  |  | / | 2.59 (1.24-5.44)^*^ |
|  | Q13: Food type changes |  | 4.60 (1.82-11.64)^**^ | 4.71 (1.50-14.76)^**^ |  |  | / | / |
|  | Age | NA | NA | 1.13 (1.09-1.18)^***^ |  | NA | NA | 1.16 (1.12-1.20)^***^ |
|  | Smoking status |  |  | 2.03 (1.26-3.27)^**^ |  |  |  | 1.66 (1.06-2.59)^*^ |
| **AUROC (95%CI)** | | 0.809 (0.763-0.848) | 0.922 (0.894-0.947) | 0.957 (0.942-0.971) |  | 0.774 (0.732-0.815) | 0.851 (0.818-0.881) | 0.935 (0.913-0.955) |
| **Sensitivity (95%CI)** | | 0.785 (0.704-0.856) | 0.862 (0.800-0.919) | 0.908 (0.856-0.955) |  | 0.623 (0.560-0.685) | 0.771 (0.715-0.822) | 0.831 (0.781-0.877) |
| **Specificity (95%CI)** | | 0.690 (0.651-0.734) | 0.846 (0.814-0.881) | 0.878 (0.847-0.906) |  | 0.806 (0.762-0.845) | 0.773 (0.723-0.820) | 0.894 (0.860-0.925) |
| **Accuracy (95%CI)** | | 0.712 (0.677-0.749) | 0.850 (0.820-0.880) | 0.885 (0.859-0.912) |  | 0.730 (0.696-0.765) | 0.772 (0.739-0.807) | 0.867 (0.839-0.894) |

*Note*: The reference category for self-reported aMMP-8 is negative self-reports; the reference category for Q1, Q3a, Q4, Q5, Q6, Q8, Q9, Q13 is the combination of “no” + “don’t know” answers; the reference category for Q2 is the combination of answers “excellent” + “very good” + “good”; the reference category for Q10 is the combination of answers “very often” + “fairly often” + “occasionally”.

LASSO: least absolute shrinkage and selection operator; aMMP-8: activated matrix metalloproteinase-8; NTP: the number of teeth present; 95% CI, confidence interval of 95%; AUROC: area under the receiver operating characteristic curve; NA: not applicable.

^***^*p* < 0.001; ^**^*p* < 0.01; ^*^*p* < 0.05.
